# Genomic Characterisation of a Novel Avipoxvirus Isolated from an Endangered Yellow-Eyed Penguin (*Megadyptes antipodes)*

**DOI:** 10.3390/v13020194

**Published:** 2021-01-28

**Authors:** Subir Sarker, Ajani Athukorala, Timothy R. Bowden, David B. Boyle

**Affiliations:** 1 Department of Physiology, Anatomy and Microbiology, School of Life Sciences, La Trobe University, Melbourne, VIC 3086, Australia; a.athukorala@latrobe.edu.au; 2 CSIRO Livestock Industries, Australian Animal Health Laboratory, Geelong, VIC 3220, Australia; timothy.bowden@csiro.au (T.R.B.); davidboyle48@gmail.com (D.B.B.)

**Keywords:** endangered yellow-eyed penguin, avipoxvirus, penguinpox virus 2, complete genome, evolution

## Abstract

Emerging viral diseases have become a significant concern due to their potential consequences for animal and environmental health. Over the past few decades, it has become clear that viruses emerging in wildlife may pose a major threat to vulnerable or endangered species. Diphtheritic stomatitis, likely to be caused by an avipoxvirus, has been recognised as a significant cause of mortality for the endangered yellow-eyed penguin (*Megadyptes antipodes*) in New Zealand. However, the avipoxvirus that infects yellow-eyed penguins has remained uncharacterised. Here, we report the complete genome of a novel avipoxvirus, penguinpox virus 2 (PEPV2), which was derived from a virus isolate obtained from a skin lesion of a yellow-eyed penguin. The PEPV2 genome is 349.8 kbp in length and contains 327 predicted genes; five of these genes were found to be unique, while a further two genes were absent compared to shearwaterpox virus 2 (SWPV2). In comparison with penguinpox virus (PEPV) isolated from an African penguin, there was a lack of conservation within the central region of the genome. Subsequent phylogenetic analyses of the PEPV2 genome positioned it within a distinct subclade comprising the recently isolated avipoxvirus genome sequences from shearwater, canary, and magpie bird species, and demonstrated a high degree of sequence similarity with SWPV2 (96.27%). This is the first reported genome sequence of PEPV2 from a yellow-eyed penguin and will help to track the evolution of avipoxvirus infections in this rare and endangered species.

## 1. Introduction

The yellow-eyed penguin, or hoiho or tarakaka (*Megadyptes antipodes*), is a species of penguin that is only found in New Zealand territories. It is one of the world’s rarest penguins, and the only extant member of the genus *Megadyptes* [1]. The species is currently listed as endangered under the International Union for Conservation of Nature (IUCN) Red List and ranked as Category B for conservation priority because of its restricted geographic range and the continuing decline in numbers of mature adults [2]. Over the last few decades, there was a serious population decline and this is projected to continue [3]. The main threats to these penguins are the destruction of breeding habitats and the predation of adults and chicks by dogs (*Canis lupus familiaris*), cats (*Felis catus*), and mustelids [4]. Moreover, a recent study described a seasonal disease (diphtheritic stomatitis) which is thought to be caused by an avipoxvirus. It is also suspected that avipoxvirus has a significant role in causing mortality in yellow-eyed penguin chicks in breeding areas of both the lower South Island and southern offshore islands of New Zealand [2].

Avipoxviruses are large, double-stranded DNA (dsDNA) viruses comprising the genus *Avipoxvirus* in the subfamily *Chordopoxvirinae*, family *Poxviridae*. Avipoxviruses are known to affect more than 329 avian species across 76 families and 20 orders of wild and domestic bird species globally [5,6,7], with many more bird species likely to be considered susceptible, which poses a risk to the health of both domesticated and wild birds. The behaviour of wild birds allows avian poxviruses to reach new hosts through bird migration, species introductions, and habitat change. Like other poxviruses, avipoxviruses can be transmitted mechanically through mosquito vectors [8]. Moreover, studies have reported that biting black flies (*Austrosimulium* sp.) are common around nest sites, and these insect vectors are thought to be responsible for transmitting *Leucocytozoon* sp. between yellow-eyed penguin chicks [9]. These insect vectors may play a role in the transmission of avipoxviruses. Avipoxviruses have been identified as an important risk factor in the conservation of small and endangered bird populations [10,11]. In affected birds, avipoxvirus infection can cause two different forms of the disease, defined as cutaneous or diphtheritic. The most common cutaneous fowlpox is characterised by proliferative ‘wart-like’ lesions that are commonly restricted to the eyes, beak, or unfeathered skin of the body. The diphtheritic form is characterised by proliferative lesions on the mucous membranes of the upper alimentary and respiratory tracts [6,12,13].

A recent study reported evidence of a potential poxvirus infection associated with diphtheritic stomatitis in the endangered yellow-eyed penguin [2]. However, the genetic and evolutionary relationships of poxviruses circulating in yellow-eyed penguins, a native species of New Zealand, are largely unknown due to the lack of sequence data. To the best of our knowledge, there are no sequences associated with avipoxvirus infections in yellow-eyed penguins in publicly available databases. Therefore, the aim of the present study was to characterise the genome sequence of PEPV2 from a yellow-eyed penguin (*M. antipodes*), sourced from New Zealand in 1997.

## 2. Materials and Methods

### 2.1. Sequencing and Assembly

Cutaneous pox lesions were collected from an endangered yellow-eyed penguin (*Megadyptes antipodes*) in 1997 by Wallaceville Animal Research Centre, New Zealand, and sent to the Australian Animal Health Laboratory, Geelong, Victoria, Australia. Subsequently, tissue homogenate was prepared and cultured in chorioallantoic membrane (CAM) and or chicken embryo cells. DNA was extracted from the virus cultured in CAM, and sequencing undertaken using TruSeq (Illumina, San Diego, CA, USA) protocols and standard multiplex adaptors available in March 2011. A paired-end 100-base-read protocol was used for sequencing on an Illumina GAIIx instrument using a previously established protocol [14]. The resulting 4,592,654 paired-end raw sequence reads were used to assemble the complete genome of PEPV2, as described previously [11,15,16,17], using CLC Genomics Workbench (version 9.5.4) and Geneious (version 10.2.2, Biomatters, New Zealand). Briefly, the sequences were processed to remove Illumina adapters, low quality reads, and ambiguous bases. Trimmed sequence reads were mapped against the chicken genome (*Gallus gallus*, GenBank accession number NC_006088) to remove any likely host DNA contamination. In addition, reads were further mapped to *Escherichia coli* bacterial genomic sequence (GenBank accession no. U00096) to remove possible bacterial contamination. Unmapped reads were used as input data for de novo assembly using CLC Genomics Workbench (version 9.5.4). This resulted in the generation of a 349,821 bp genome. Clean raw reads were mapped back to the assembled PEPV2 genome and resulted in an average coverage of 560.61x.

### 2.2. Genome Annotations

The assembled genome of PEPV2 was annotated using Geneious (version 10.2.2) software, and further verification of the predicted ORFs was performed using CLC Genomic Workbench (version 9.5.4). According to Sarker, et al. [11], open reading frames (ORFs) longer than 50 amino acids, with a methionine start codon (ATG) and minimal overlap with other ORFs (not exceeding 50% of one of the genes), were selected and annotated. ORFs shorter than 50 amino acids that had previously been annotated in other poxvirus genomes were also included. These ORFs were subsequently extracted into a FASTA file, and similarity BLAST searches were performed. Predicted ORFs that showed significant sequence similarity to known viral or cellular genes (BLAST E value ≤ 10^−5^) were annotated as potential genes [18]. To identify the likely promoter sequences of predicted ORFs of PEPV2, a promoter motif search analysis was conducted using CLC Genomic Workbench (version 9.5.4), where vaccinia virus unique promoter sequences were used [19,20,21,22].

To predict the function of unique ORFs tentatively identified in this study, the derived protein sequence of each ORF was searched by multiple applications to identify conserved domains or motifs. Transmembrane helices were searched using the TMHMM package (version 2.0) [23], HMMTOP [24], TMpred [25], and Geneious (version 10.2.2). Additionally, searches for conserved secondary structure (HHpred) [26] and protein homologues, using Phyre2 [27] and SWISS-MODEL [28], were used to predict the function of unique ORFs identified in this study.

### 2.3. Comparative Genomics

Genomic features of the newly sequenced PEPV2 were visualised using Geneious (version 10.2.2). Sequence similarity percentages between representative chordopoxvirus (ChPV) and PEPV2 complete genome sequences were determined using tools available in Geneious (version 10.2.2). Dot plots were created based on the EMBOSS dottup program in Geneious software, with word size = 12 [29].

### 2.4. Phylogenetic Analysis

For phylogenetic analysis, all available avipoxvirus genomes were downloaded from GenBank. Genome sequences of each of the fully sequenced avipoxviruses including canarypox virus (CNPV; AY318871) [30], shearwaterpox virus 1 (SWPV1; KX857216) and 2 (SWPV2; KX857215) [11], pigeonpox virus (FeP2; KJ801920) [31], fowlpox virus (FWPV; AF198100, MF766430-32, MH709124-25, MH719203, MH734528 and AJ581527) [32,33,34], turkeypox virus (TKPV; NC_028238) [35], penguinpox virus (PEPV; KJ859677) [31], flamingopox virus (FGPV; MF678796) [7], magpiepox virus (MPPV; MK903864) [16], and mudlarkpox virus (MLPV; MT978051) [36] were downloaded from GenBank and used in further analysis of penguinpox virus 2 (PEPV2; MW296038). Nucleotide sequences of the partial DNA polymerase and partial p4b genes as well as concatenated amino acid sequences of the selected nine poxvirus core proteins (RNA polymerase subunit RPO132, RNA polymerase subunit RPO147, mRNA capping enzyme large subunit, RNA polymerase-associated protein RAP94, virion core protein P4a, virion core protein P4b, early transcription factor large subunit VETFL, NTPase, and DNA polymerase) were aligned using the MAFTT L-INS-I algorithm implemented in Geneious (version 7.388) [37]. To determine the best-fit model to compute phylogenetic analyses, a model test was performed using CLC Genomics Workbench (version 9.5.4), which favoured a general time-reversible model with gamma distribution rate variation and a proportion of invariable sites (GTR+G+T). Phylogenetic analyses for nucleotide sequences were performed using the GTR substitution model with 1000 bootstrap support in CLC Genomics Workbench (version 9.5.4), but the LG substitution model was chosen for concatenated amino acid sequences in Geneious (version 10.2.2).

## 3. Results

### 3.1. Genome of PEPV2

The complete genome sequence of PEPV2 was a linear double-stranded DNA molecule 349,821 bp in length, and was submitted to GenBank under accession number MW296038. Like most other avipoxviruses [11,32,33], the PEPV2 genome comprised a large central coding region bounded by two identical inverted terminal repeats (ITRs) of 4054 bp each (coordinates 1–4054 sense and 345,768–349,821 antisense orientation). The PEPV2 genome sequenced in this study showed the highest (96.27%) nucleotide identity with the SWPV2 genome sequenced from the wedge-tailed shearwater (*Ardenna pacifica*) bird in Australia (Table 1) (GenBank accession no. KX857215) [11]. In comparison with another penguinpox virus (PEPV; GenBank accession number KJ859677) sequenced from an African penguin [31], the penguinpox virus 2 sequenced in this study was significantly different (showing only 50% nucleotide identity). The A+T content of the PEPV2 genome was >69%, which shared greatest similarity with other sequenced avipoxviruses from passerine and shearwater bird species (Table 1).

### 3.2. Genome Annotation and Comparative Analyses of PEPV2

**Figure 1:**
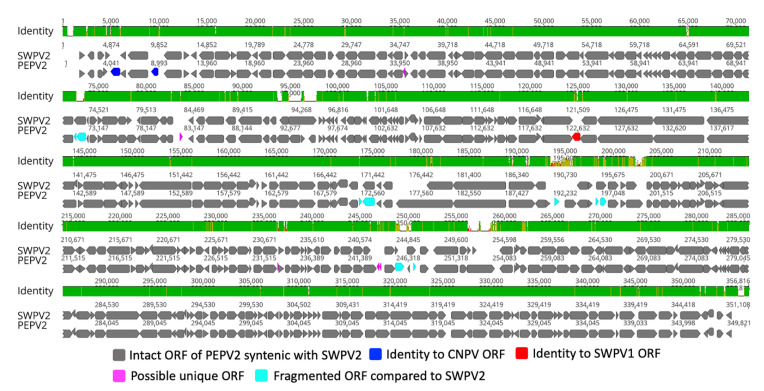
Genomic illustration of the novel PEPV2. The arrows depict the direction of transcription of genes and open reading frames (ORFs). Each gene or ORF is colour coded, as indicated by the key in the legend. The top graph represents the mean pairwise sequence identity over all pairs in the column between PEPV2 and SWPV2 (green: 100% identity; mustard: ≥30% and <100% identity; red: <30% identity).

The PEPV2 genome contained 327 predicted methionine-initiated ORFs encoding proteins ranging from 45 to 1936 amino acids in length that have been annotated as putative genes and numbered from left to right (Figure 1 and Supplementary Table S1), of which four ORFs were located within the inverted terminal repeats (ITRs) and were therefore present as diploid copies. Comparative analysis of the predicted ORF sequences was performed, and a significant number of ORFs (322) were found to be homologues with other ChPV gene products (Supplementary Table S1). Among these conserved ChPV gene products, the highest number of protein-coding genes (319) in PEPV2 were homologues to the recently isolated SWPV2. The remaining two gene products (PEPV2-005 and -008) were homologous to ORFs of CNPV, and a further gene product was a homologue to SWPV1 (PEPV2-124) (Supplementary Table S1). All conserved genes of PEPV2 showed the highest sequence similarity to homologue of the isolated SWPV2 and CNPV, and these observations imply that the conserved PEPV2 genes share a common evolutionary history with the poxviruses infecting Pacific shearwaters and canary bird species [11,30]. In comparison to SWPV2, two gene products (SWPV2-121 and -213) were not represented in the PEPV2 genome, and a further six genes were found to be truncated/fragmented (Figure 1 and Supplementary Table S1).

Interestingly, PEPV2 contained five predicted protein-coding genes (ORF029, -077, -207, -220 and -221) that were not present in any other poxvirus, nor did they match any sequences in the NR protein database using BLASTX and BLASTP; these unique ORFs encoded proteins of 51 to 89 amino acids in length (Supplementary Table S1). Furthermore, except for PEPV2-ORF077, each of these unique protein-coding genes was predicted to contain a single transmembrane helix (TMH) using at least two of the software packages employed in this study. However, we did not find any significant homology with known proteins for the unique ORFs encoded in the PEPV2 genome when using Phyre2, HHpred, and SWISS-MODEL, which might be due to the lack of closely related structures in the database.

**Figure 2:**
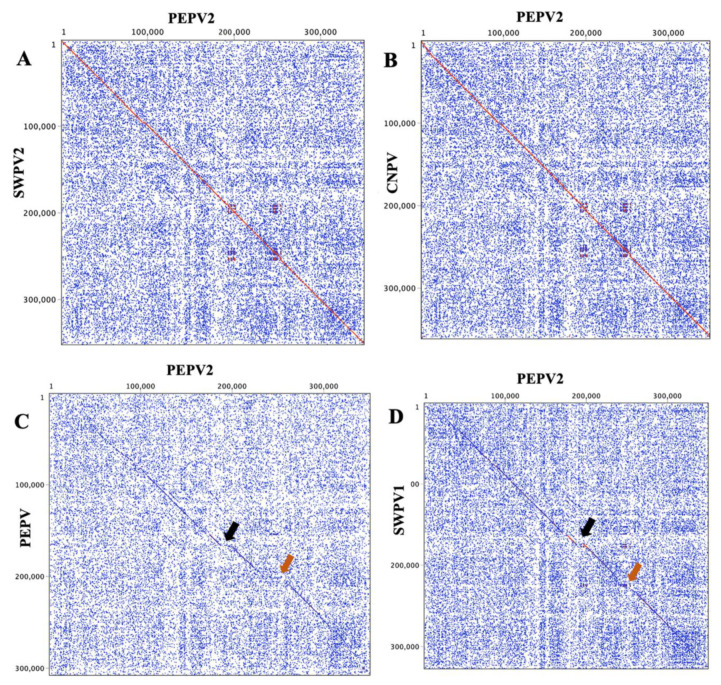
Dot plots of the PEPV2 genome (x-axis) vs. other sequenced avipoxvirus genomes (y-axis). (**A**) PEPV2 vs. SWPV2, (**B**) PEPV2 vs. CNPV, (**C**) PEPV2 vs. PEPV, and (**D**) PEPV2 vs. SWPV1. The Classic colour scheme was chosen in Geneious (version 10.2.2) for the dot plot lines according to the length of the match, from blue for short matches to red for matches over 100 bp long. Black arrows indicate the first region of major difference (PEPV2-158 to -170) and orange arrows indicate the second region of major difference (PEPV2-214 to -236). Window size = 12.

Comparison of the PEPV2 genome to that of another penguinpox virus (PEPV) genome isolated from an African penguin (*Spheniscus demersus*) showed major differences that were most evident within three distinct regions (Figure 2). The first region was within the viral ITRs, which was consistent with other ChPVs [15,38,39,40], and the other two regions were within the central portion of the genome (Figure 2C). The second region was flanked by PEPV2-158 and -170 (Figure 2C; black arrow). Within this region, multiple SNPs and insertions/deletions (indels) led to variation in most of the ORFs predicted in the PEPV2 genome. In comparison to PEPV, the PEPV2 genome encoded some additional ORFs that mostly belonged to multigene families including TGF beta-like protein, N1R/p28-like protein, Ig-like domain protein and thymidylate kinase. The third region was flanked by PEPV2-214 and -236 (Figure 2C; orange arrow), and was also likely to represent some additional ORFs. Compared to PEPV, a large number of ORFs in the PEPV2 genome encoded proteins that also mainly belonged to multigene families such as the N1R/p28-like protein, ankyrin repeat protein, deoxycytidine kinase-like protein, vaccinia C4L/C10L-like protein, CC chemokine-like protein, and hypothetical protein.

### 3.3. Gene Promoter Motif Elements

Among the predicted 327 ORFs of PEPV2, only 139 ORFs showed homologues to vaccinia virus (VACV-Cop) [21] (Supplementary Table S1); however, the protein identities of individual ORFs were very low. Among the predicted 40 ORFs of PEPV2 that were found to be homologues to VACV, only 13 ORFs contained a poxvirus early transcriptional stop sequence (TTTTTXT, where X is any nucleotide) near the translational stop codon (50 bases upstream to 100 bases downstream). Further 57 ORFs of PEPV2 also contained a poxvirus early transcriptional stop sequence. As seen in other poxviruses, many genes with potential early promoters are members of gene families or putative host range genes or both (Supplementary Table S1). Two of the ORFs of PEPV2 that were homologues of VACV intermediate genes (PEPV2-069 and -071) contain the VACV intermediate promoter sequence (AAAXAAX_11–13_TAAA) [19,41,42]. A total of 28 putative late PEPV2 ORFs (Supplementary Table S1), contain the VACV late-promoter sequence (TAAATG) at ATG codon [43]. Moreover, the TAAATG late promoter of five of the putative late PEPV2 ORFs was shown to be located upstream of PEPV2 late genes, which is consistent with the previous findings in other poxviruses [32,44,45,46].

### 3.4. Evolutionary Relationships of PEPV2

**Figure 3:**
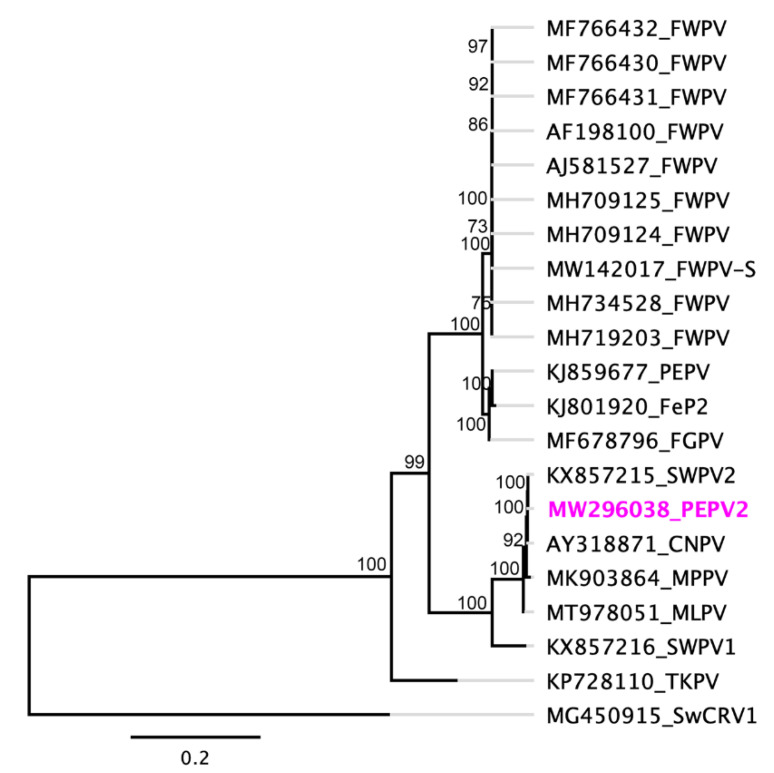
Phylogenetic relationship between PEPV2 and other chordopoxviruses. A maximum likelihood (ML) tree was constructed from multiple alignments of the concatenated amino acid sequences of the selected nine poxvirus core proteins using Geneious (version 10.2.2). The numbers on the left show bootstrap values as percentages. The labels were aligned in the ML tree. The labels at branch tips refer to original ChPV GenBank accession numbers followed by abbreviated species names. The following abbreviations and GenBank accession details for the selected ChPVs used are: penguinpox virus 2 (PEPV2; MW296038), fowlpox virus standard vaccine strain (FWPV-S; MW142017), canarypox virus (CNPV; AY318871), pigeonpox virus (FeP2; KJ801920), penguinpox virus (PEPV; KJ859677), fowlpox virus (FWPV; AF198100, MF766430-32, MH709124-25, MH719203, MH734528, and AJ581527), fowlpox virus Australian vaccine strain (FWPV-S; MW142017) [48], shearwaterpox virus 1 (SWPV1; KX857216), shearwaterpox virus 2 (SWPV2; KX857215), turkeypox virus (TKPV; NC_028238); flamingopox virus (FGPV; MF678796), magpiepox virus (MPPV; MK903864), and mudlarkpox virus (MLPV; MT978051). Saltwater crocodile poxvirus (SwCRV1; MG450915) [38] was used as an outgroup. The novel PEPV2 is shown in bold font and pink text.

Phylogenetic analysis based on the concatenated amino acid sequences of the selected nine core poxvirus proteins supported the inclusion of the newly assembled PEPV2 in the genus *Avipoxvirus*. In the resulting maximum likelihood (ML) tree, the novel PEPV2 was located within the same subclade as CNPV, SWPV, MPPV, and MLPV with strong bootstrap support (100%) (Figure 3), suggesting that it may represent an ancient evolutionary lineage within the genus. However, the novel PEPV2 was positioned in a separate clade from another penguinpox virus (PEPV) that was isolated from an African penguin (*Spheniscus demersus*) [31]. Using the same set of concatenated protein sequences, we found that the maximum inter-lineage sequence identity values between the novel PEPV2 and other avipoxviruses were >82.0% (PEPV2 vs. PEPV) and >99.0% (PEPV2 vs. SWPV2 or CNPV), which mirrored the phylogenetic position of this novel avipoxvirus sequenced from an endangered yellow-eyed penguin. A greater selection of poxviruses was included in the phylogenetic tree with partial nucleotide sequences from the DNA polymerase gene (Supplementary Figure S1) and p4b gene (Supplementary Figure S2). We discovered that several other avipoxviruses were represented within the PEPV2, CNPV, SWPV2, MPPV, and MLPV clade. This included a poxvirus isolated from a common bullfinch (*Pyrrhula pyrrhula*) in Belgium [47] and a northern harrier (*Circus cyaneus*) in Spain [47], which is almost identical to PEPV2 within this relatively small fragment of the genome.

## 4. Discussion

This study reports the characterisation of the complete genome sequence of a novel avipoxvirus, PEPV2, isolated from cutaneous pox lesions in a yellow-eyed penguin. Since its initial recognition, diphtheritic stomatitis, likely caused by an avipoxvirus, has been associated with high mortality in yellow-eyed penguin chicks in breeding areas of both the lower South Island and southern offshore islands of New Zealand [2]. In the absence of any in vivo or in vitro experiments, the present study was unbale to directly link the isolated PEPV2 to diphtheritic stomatitis. A recent study reported evidence of avipoxvirus viral DNA in the early oral lesions of yellow-eyed penguins by PCR and it was suspected to be an etiological agent of diphtheritic stomatitis; however, there no sequence data were made available [2]. Consequently, no taxonomic classification has been granted for PEPV2 by the International Committee on Taxonomy of Viruses (ICTV; https://talk.ictvonline.org/taxonomy/) [49], and no phylogenetic relationship has been established with other members of the avipoxviruses. These facts accentuate the importance of this study in characterising a likely poxvirus infection that may add to the morbidity burden in the endangered yellow-eyed penguin.

After examining the phylogenetic relationship between the novel PEPV2 and other avipoxviruses, we found that PEPV2 was most closely related to SWPV2, CNPV, and MPPV. This may indicate that these avipoxviruses originated from a common ancestor that diverged from an SWPV-like progenitor [11]. However, the PEPV2 genome sequenced from a yellow-eyed penguin sourced from New Zealand was distantly related with a previously isolated penguinpox virus (PEPV) from an African penguin [31], which may indicate that these two penguin species were likely infected with two different species of avipoxvirus. Well-supported phylogenetic trees were also produced using both the partial nucleotide sequences of the DNA polymerase and p4b genes, and they showed that the PEPV2 isolated in this study was located within a subclade (B1) (Supplementary Figures S1 and S2) consisting of SWPV2, CNPV, and MPPV, with the addition of a large number of avipoxviruses isolated globally. These results further conclude that PEPV2 may be more closely related to other avipoxviruses sourced from various bird species at a conserved gene level. However, considering the narrow genetic diversity and geographic distribution of yellow-eyed penguins, it is perhaps not surprising that this species may be exposed to this novel PEPV2 infection.

Some aspects of avipoxvirus transmission in this naïve host species are difficult to explain fully without conducting virus-transmission experiments. However, biting black flies (*Austrosimulium* sp.) are common around nest sites, and these insect vectors are thought to be responsible for transmitting *Leucocytozoon* sp. between yellow-eyed penguin chicks [9]. In addition, mosquitoes are thought to play a role in the mechanical transmission of avipoxviruses within the wild bird population [8]. It would therefore seem likely that, as for other avipoxviruses, transmission of PEPV2 in the yellow-eyed penguin is also mediated by insect vectors.

## 5. Conclusions

This study reports the discovery and genomic characterisation of the first avipoxvirus, PEPV2, isolated from an endangered yellow-eyed penguin. The novel PEPV2 genome was highly divergent when compared to previously sequenced avipoxviruses from an African penguin and other avian species. Considering the overall genome architecture, PEPV2 appears to represent a novel species within the genus *Avipoxvirus*, family *Poxviridae*. Characterisation of the novel PEPV2 genome will contribute to a better understanding of avipoxvirus diversity and evolution. Obtaining and sequencing additional poxvirus isolates, including from oral lesions in yellow-eyed penguins with diphtheritic stomatitis, will also be important to further investigate the pathogenesis of PEPV2 and host specificity of avipoxvirus infections in this endangered bird species.

## Figures and Tables

**Table 1:** Comparative analysis of representative avipoxviruses and PEPV2 based on complete genome nucleotide sequences.

Avipoxvirus	Genome Identity (%)	A+T Content (%)	Number of ORFs	References
MW296038_PEPV2		69.9	327	This study
KX857215_SWPV2	96.27	69.8	312	[11]
KX857216_SWPV1	61.79	72.4	310	[11]
MT978051_MLPV	89.34	70.2	328	[36]
MK903864_MPPV	79.14	70.4	352	[16]
AY318871_CNPV	93.23	69.6	328	[30]
KJ801920_FeP2	47.73	70.5	271	[31]
KJ859677_PEPV	50.06	70.5	285	[31]
MF678796_FGPV	46.70	70.5	285	[7]
KP728110_TKPV	33.60	70.2	171	[35]
AF198100_FWPV	49.11	69.1	260	[32]

## Data Availability

The complete genome sequence and associated datasets generated during this study were deposited in GenBank under the accession number MW296038.

## Supplementary Materials

The following are available online at https://www.mdpi.com/1999-4915/13/2/194/s1, Figure S1. Maximum likelihood phylogenetic tree from partial nucleotide sequences of the DNA polymerase gene of selected avipoxviruses. Figure S2. Maximum likelihood phylogenetic tree from partial nucleotide sequences of the p4b gene of selected avipoxviruses. Table S1. Penguinpox virus 2 (PEPV2) genome annotations and comparative analysis.
