# Supplementary material for: Genomic Characterisation of a Novel Avipoxvirus Isolated from an Endangered Yellow-Eyed Penguin (Megadyptes antipodes)
Source: Viruses. 2021 Jan 28;13(2):194. doi: 10.3390/v13020194 (PMC7911368; doi:10.3390/v13020194)
Supplement: Supplementary file 1 [file viruses-13-00194-s001.pdf]

viruses

Supplementary File

Genomic characterisation of a novel avipoxvirus from an endangered yellow-eyed penguin (*Megadyptes antipodes*)

Subir Sarker[1]*, Ajani Athukorala[1], Timothy R. Bowden[2,3] and David B. Boyle[2]

[1] Department of Physiology, Anatomy and Microbiology, School of Life Sciences, La Trobe University, Melbourne, VIC 3086, Australia. (S.S. email: S.Sarker@latrobe.edu.au; A.A. email: a.athukorala@latrobe.edu.au).
[2] CSIRO Livestock Industries, Australian Animal Health Laboratory, Geelong, VIC 3220, Australia. (T.R.B. email: timothy.bowden@csiro.au; D.B.B email: davidboyle48@gmail.com).
[3] Present address: CSIRO Australian Animal Health Laboratory, Australian Centre for Disease Preparedness, Geelong, VIC 3220, Australia. (T.R.B. email: timothy.bowden@csiro.au)

* Correspondence: S.Sarker@latrobe.edu.au; Tel.: +61 3 9479 2317; fax: (+61) 3 9479 1222

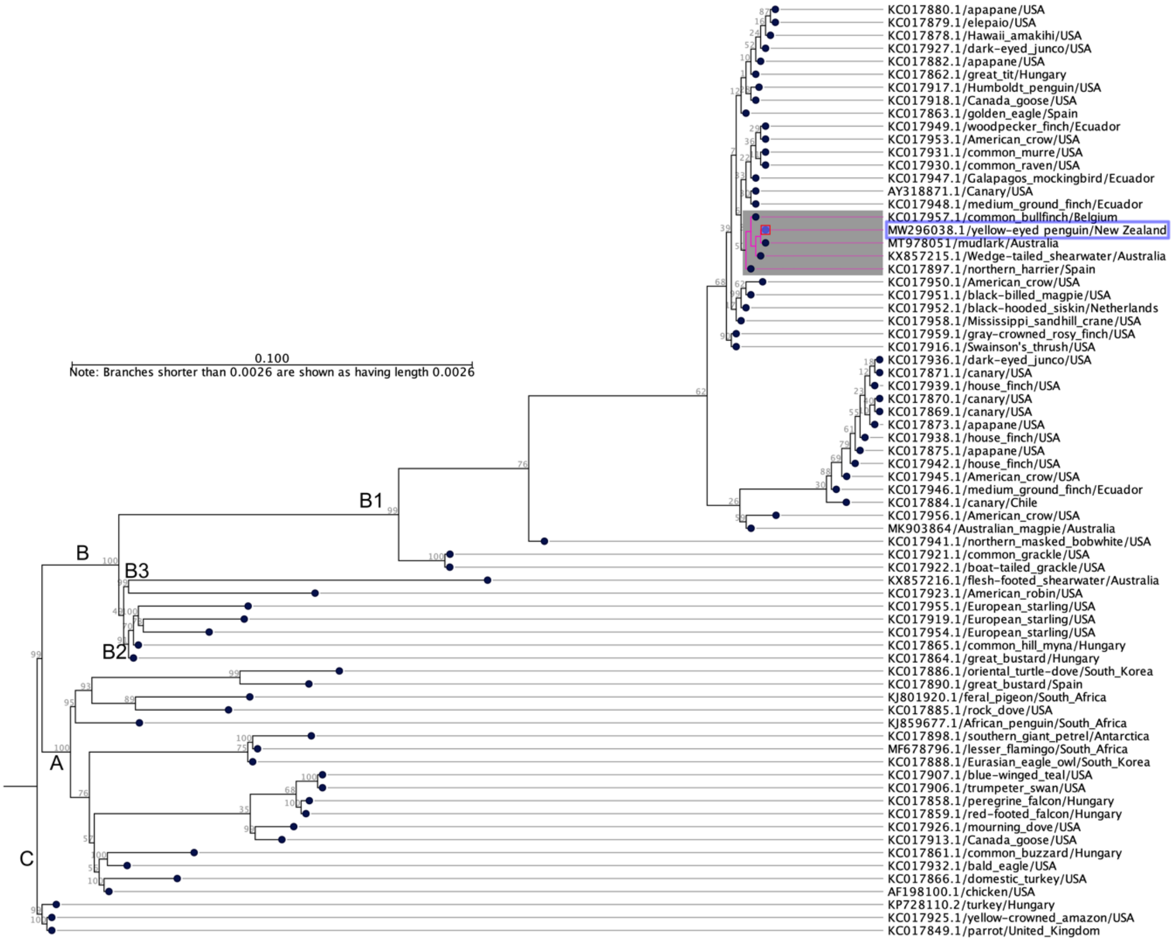

Figure S1. Maximum Likelihood phylogenetic tree from partial nucleotide sequences of the DNA polymerase gene of selected avipoxviruses. Labels at branch tips refer to GenBank accession number/species/country of origin. The numbers on the left show bootstrap values as percentages and PEPV2 is highlighted using a blue box. The ML tree was displayed as a phylogram. The bootstrap value assigned to a node in the output tree is the percentage (0-100) of the bootstrap resamples which resulted in a tree containing the same subtree as that rooted at the node. According to Gyuranecz, *et al.* [47], major clades were designated A, B and C, where B1-B3 represent subclades. The subclade line relevant to PEPV2 is highlighted with grey colour background.

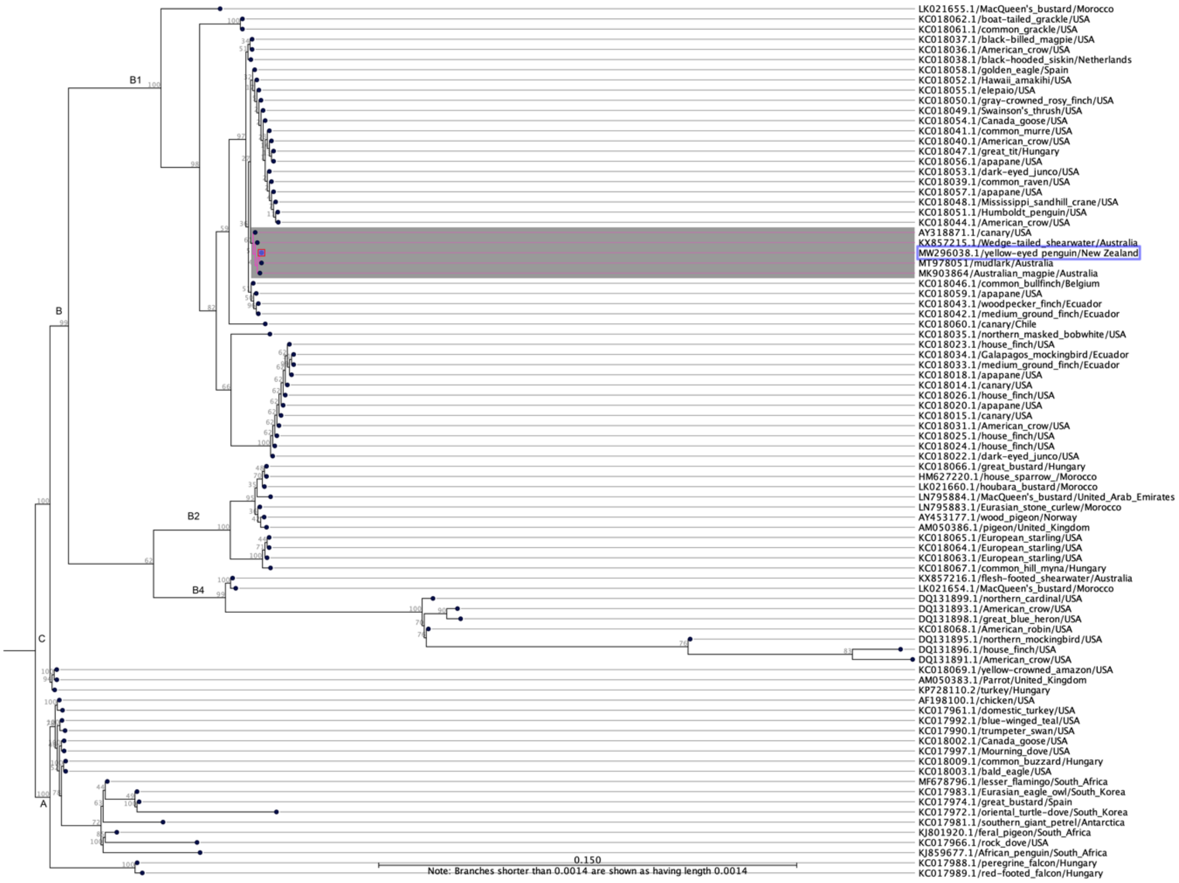

Figure S2. Maximum likelihood phylogenetic tree from partial nucleotide sequences of the p4b gene of selected avipoxviruses. Labels at branch tips refer to GenBank accession number/species/country of origin. The numbers on the left show bootstrap values as percentages and PEPV2 is highlighted using a blue box. The ML tree was displayed as a phylogram. The bootstrap value assigned to a node in the output tree is the percentage (0-100) of the bootstrap resamples which resulted in a tree containing the same subtree as that rooted at the node. According to Gyuranecz, *et al.* [47], major clades were designated A, B and C, where B1-B4 represent subclades. The subclade line relevant to PEPV2 is highlighted with grey colour background.

Supplementary Table S1. Penguinpox virus 2 (PEPV2) genome annotations and comparative analysis

PEPV2 synteny	PEPV2 genome coordinates	SWPV2 synteny	PEPV2 AA size	SWPV2 AA size	SWPV2 BLAST hits	PEPV2 AA identity (%)	PEPV2 AA identity compare to VACV-Cop (%)	VACV-Cop synteny	Predicted promoter in VACV-Cop	Predicted promoter in PEPV2	notes
PEPV2-001	830-1345	SWPV2-001	171	171	SWPV2-001 hypothetical protein	100				E	identical to PEPV2-327
PEPV2-002	2274-1648	SWPV2-002	208	208	SWPV2-002 C-type lectin-like protein	100				E	identical to PEPV2-326
PEPV2-003	2682-3350	SWPV2-003	222	222	SWPV2-003 conserved hypothetical protein	100					identical to PEPV2-325
PEPV2-004	3286-3648	SWPV2-004	120	134	SWPV2-004 conserved hypothetical protein	70.83					identical to PEPV2-324
PEPV2-005	4966-4046		306			97.70					CNPV007 ankyrin repeat protein
PEPV2-006	5522-5013	SWPV2-005	169	169	SWPV2-005 C-type lectin-like protein	100				E	
PEPV2-007	7876-5810	SWPV2-006	688	688	SWPV2-006 ankyrin repeat protein	100	23.91	B4R	L		
PEPV2-008	8895-8251		214			99.53					CNPV010 ankyrin repeat protein
PEPV2-009	11285-9525	SWPV2-007	586	586	SWPV2-007 ankyrin repeat protein	100	28.83	M1L	E	E	
PEPV2-010	11550-12119	SWPV2-008	189	189	SWPV2-008 conserved hypothetical protein	100					
PEPV2-011	12832-12326	SWPV2-009	168	168	SWPV2-009 conserved hypothetical protein	100					
PEPV2-012	14622-13150	SWPV2-010	490	490	SWPV2-010 Ig-like domain protein	100					
PEPV2-013	14786-16435	SWPV2-011	549	528	SWPV2-011 ankyrin repeat protein	96.17	28.72	M1L	E		
PEPV2-014	16496-17002	SWPV2-012	168	168	SWPV2-012 C-type lectin-like protein	100	23.30	A40R	E	E	
PEPV2-015	17106-18545	SWPV2-013	479	479	SWPV2-013 ankyrin repeat protein	100	27.59	B4R	L	E	
PEPV2-016	19216-18644	SWPV2-014	190	190	SWPV2-014 IL-10-like protein	100				E	
PEPV2-017	20643-19333	SWPV2-015	436	436	SWPV2-015 ankyrin repeat protein	100	27.54	M1L	E	E	
PEPV2-018	20834-22093	SWPV2-016	419	419	SWPV2-016 ankyrin repeat protein	100	38.24	M1L	E	E	
PEPV2-019	23836-22229	SWPV2-017	535	535	SWPV2-017 ankyrin repeat protein	100	21.53	B4R	L	E	
PEPV2-020	24953-23877	SWPV2-018	358	358	SWPV2-018 putative serpin	100	27.91	C12L	E		
PEPV2-021	26309-25035	SWPV2-019	424	424	SWPV2-019 vaccinia C4L/C10L-like protein	100					
PEPV2-022	26587-27123	SWPV2-020	178	178	SWPV2-020 hypothetical protein	100					

PEPV2 synteny	PEPV2 genome coordinates	SWPV2 synteny	PEPV2 AA size	SWPV2 AA size	SWPV2 BLAST hits	PEPV2 AA identity (%)	PEPV2 AA identity compare to VACV-Cop (%)	VACV-Cop synteny	Predicted promoter in VACV-Cop	Predicted promoter in PEPV2	notes
PEPV2-023	28239-27337	SWPV2-021	300	300	SWPV2-021 alpha-SNAP-like protein	100					
PEPV2-024	29510-28362	SWPV2-022	382	382	SWPV2-022 ankyrin repeat protein	100	21.22	C9L	E		
PEPV2-025	31459-29579	SWPV2-023	626	626	SWPV2-023 ankyrin repeat protein	100				E	
PEPV2-026	32675-31578	SWPV2-024	365	365	SWPV2-024 ankyrin repeat protein	100					
PEPV2-027	33213-32785	SWPV2-025	142	142	SWPV2-025 C-type lectin-like protein	100				E	
PEPV2-028	34291-33269	SWPV2-026	340	340	SWPV2-026 ankyrin repeat protein	100	26.83	B4R	L	E	
PEPV2-029	34554-34354		66							E	hypothetical protein, unique to PEPV2, detected 1 transmembrane helix
PEPV2-030	34534-34893	SWPV2-027	119	119	SWPV2-027 hypothetical protein	100				E/L	
PEPV2-031	35849-35121	SWPV2-028	242	242	SWPV2-028 Ig-like domain putative IFN-gamma binding protein	100				E	
PEPV2-032	36665-35925	SWPV2-029	246	246	SWPV2-029 Ig-like domain protein	100				E	
PEPV2-033	38740-36761	SWPV2-030	659	659	SWPV2-030 ankyrin repeat protein	100	32.50	M1L	E	E	
PEPV2-034	39459-39058	SWPV2-031	133	133	SWPV2-031 C-type lectin-like protein	100				L	
PEPV2-035	39843-39556	SWPV2-032	95	95	SWPV2-032 conserved hypothetical protein	100				E	
PEPV2-036	39907-40446	SWPV2-033	179	179	SWPV2-033 conserved hypothetical protein	100					
PEPV2-037	41692-40451	SWPV2-034	413	413	SWPV2-034 vaccinia C4L/C10L-like protein	99.76	24.34	C10L	E		
PEPV2-038	41810-42793	SWPV2-035	327	327	SWPV2-035 G protein-coupled receptor-like protein	100					
PEPV2-039	44587-42812	SWPV2-036	591	591	SWPV2-036 ankyrin repeat protein	99.66	23.15	B4R	L		
PEPV2-040	45952-44660	SWPV2-037	430	430	SWPV2-037 ankyrin repeat protein	100	26.37	B18R	E		
PEPV2-041	47818-46001	SWPV2-038	605	605	SWPV2-038 ankyrin repeat protein	100	21.60	B4R	L		
PEPV2-042	48529-47924	SWPV2-039	201	201	SWPV2-039 conserved hypothetical protein	100					
PEPV2-043	50013-48571	SWPV2-040	480	480	SWPV2-040 ankyrin repeat protein	100	25.16	B18R	E	E	
PEPV2-044	50279-51277	SWPV2-041	332	332	SWPV2-041 G protein-coupled receptor-like protein	100					

PEPV2 synteny	PEPV2 genome coordinates	SWPV2 synteny	PEPV2 AA size	SWPV2 AA size	SWPV2 BLAST hits	PEPV2 AA identity (%)	PEPV2 AA identity compare to VACV-Cop (%)	VACV-Cop synteny	Predicted promoter in VACV-Cop	Predicted promoter in PEPV2	notes
PEPV2-045	52656-51304	SWPV2-042	450	450	SWPV2-042 ankyrin repeat protein	100	34.09	B4R	L		
PEPV2-046	53097-52723	SWPV2-043	124	124	SWPV2-043 conserved hypothetical protein	100					
PEPV2-047	55670-53265	SWPV2-044	801	801	SWPV2-044 alkaline phosphodiesterase-like protein	100					
PEPV2-048	56210-55758	SWPV2-045	150	150	SWPV2-045 hypothetical protein	100				E	
PEPV2-049	57322-56264	SWPV2-046	352	352	SWPV2-046 ankyrin repeat protein	100					
PEPV2-050	58595-57369	SWPV2-047	408	408	SWPV2-047 DNase II-like protein	100					
PEPV2-051	59136-58621	SWPV2-048	171	171	SWPV2-048 C-type lectin-like protein	100					
PEPV2-052	59756-59316	SWPV2-049	146	146	SWPV2-049 conserved hypothetical protein	100					
PEPV2-053	60171-59749	SWPV2-050	140	140	SWPV2-050 conserved hypothetical protein	100					
PEPV2-054	60714-60223	SWPV2-051	163	163	SWPV2-051 conserved hypothetical protein	100					
PEPV2-055	61148-60711	SWPV2-052	145	145	SWPV2-052 CNLV056 dUTPase	100	52.78	F2L	E		
PEPV2-056	62095-61175	SWPV2-053	306	306	SWPV2-053 putative serpin	100					
PEPV2-057	62668-62126	SWPV2-054	180	180	SWPV2-054 bcl-2 like protein	100				E	
PEPV2-058	63741-62725	SWPV2-055	338	338	SWPV2-055 putative serpin	100				E	
PEPV2-059	64622-63810	SWPV2-056	270	206	SWPV2-056 conserved hypothetical protein	99.51				E	
PEPV2-060	66409-64712	SWPV2-057	565	565	SWPV2-057 DNA ligase	100	46.94	A50R	E/L	E	
PEPV2-061	67500-66448	SWPV2-058	350	350	SWPV2-058 putative serpin	100	25.00	K2L		E	
PEPV2-062	68647-67571	SWPV2-059	358	358	SWPV2-059 hydroxysteroid dehydrogenase-like protein	100	38.24	A44L	E	E	
PEPV2-063	69560-68709	SWPV2-060	283	283	SWPV2-060 TGF-beta-like protein	100					
PEPV2-064	71391-69640	SWPV2-061	583	583	SWPV2-061 semaphorin-like protein	100	31.66	A39R	E/L	E	
PEPV2-065	71673-71491	SWPV2-062	60	399	SWPV2-062 hypothetical protein	100					
PEPV2-066	71896-71717	SWPV2-062	59	399	SWPV2-062 hypothetical protein	100					
PEPV2-067	72151-71978	SWPV2-063	57	57	SWPV2-063 hypothetical protein	100					

viruses MDPI

PEPV2 synteny	PEPV2 genome coordinates	SWPV2 synteny	PEPV2 AA size	SWPV2 AA size	SWPV2 BLAST hits	PEPV2 AA identity (%)	PEPV2 AA identity compare to VACV-Cop (%)	VACV-Cop synteny	Predicted promoter in VACV-Cop	Predicted promoter in PEPV2	notes
PEPV2-068	72311-73084	SWPV2-064	257	257	SWPV2-064 GNS1/SUR4-like protein	100					
PEPV2-069	73177-73644	SWPV2-065	155	155	SWPV2-065 late transcription factor VLTF-2	100	47.18	A1L	E/L	I	
PEPV2-070	73661-75316	SWPV2-066	551	551	SWPV2-066 putative rifampicin resistance protein, IMV assembly	100	56.16	D13L	L	L	
PEPV2-071	75348-76217	SWPV2-067	289	289	SWPV2-067 mRNA capping enzyme small subunit	100	57.54	D12L	E/L		
PEPV2-072	76238-77179	SWPV2-068	313	132	SWPV2-068 CC chemokine-like protein	100					
PEPV2-073	77586-77257	SWPV2-069	109	109	SWPV2-069 hypothetical protein	100					
PEPV2-074	77657-79564	SWPV2-070	635	635	SWPV2-070 NPH-I, transcription termination factor	100	60.83	D11L	L	L	
PEPV2-075	80247-79561	SWPV2-071	228	228	SWPV2-071 mutT motif putative gene expression regulator	100	37.96	D10R	L		
PEPV2-076	80944-80231	SWPV2-072	237	232	SWPV2-072 mutT motif	97.89	47.22	D9R	E	E	
PEPV2-077	81692-81961		89								hypothetical protein, unique to PEPV2
PEPV2-078	83068-82586	SWPV2-073	160	160	SWPV2-073 RNA polymerase subunit RPO18	100	57.50	D7R	E/L	E	
PEPV2-079	84228-83404	SWPV2-074	274	274	SWPV2-074 Ig-like domain protein	100				E	
PEPV2-080	86252-84351	SWPV2-075	633	633	SWPV2-075 early transcription factor small subunit VETFS	99.84	72.24	D6R	L		
PEPV2-081	87457-86456	SWPV2-076	333	334	SWPV2-076 Ig-like domain protein	98.80					
PEPV2-082	90167-87783	SWPV2-077	794	794	SWPV2-077 NTPase, DNA replication	100	54.26	D5R	E/L		
PEPV2-083	90987-90322	SWPV2-078	221	221	SWPV2-078 CC chemokine-like protein	100					
PEPV2-084	91726-91070	SWPV2-079	218	218	SWPV2-079 uracil DNA glycosylase	99.54	54.17	D4R	E		
PEPV2-085	92510-91767	SWPV2-080	247	303	SWPV2-080 putative RNA phosphatase	98.72	34.09	H1L	L		
PEPV2-086	93745-92558	SWPV2-204	395	401	SWPV2-204 conserved hypothetical protein	40.05					
PEPV2-087	93830-94195	SWPV2-081	121	112	SWPV2-081 TNFR-like protein	97.06	48.72	C22L/B28R	L		
PEPV2-088	95309-94653	SWPV2-208	218	85	SWPV2-208 N1R/p28-like protein	70.39					
PEPV2-089	95672-96067	SWPV2-082	131	131	SWPV2-082 putative glutathione peroxidase	100					

**PEPV2 synteny**	**PEPV2 genome coordinates**	**SWPV2 synteny**	**PEPV2 AA size**	**SWPV2 AA size**	**SWPV2 BLAST hits**	**PEPV2 AA identity (%)**	**PEPV2 AA identity compare to VACV-Cop (%)**	**VACV-Cop synteny**	**Predicted promoter in VACV-Cop**	**Predicted promoter in PEPV2**	**notes**
PEPV2-090	96092-96394	SWPV2-083	100	100	SWPV2-083 conserved hypothetical protein	100				L	
PEPV2-091	96878-96399	SWPV2-084	159	159	SWPV2-084 conserved hypothetical protein	100					
PEPV2-092	97248-96865	SWPV2-085	127	127	SWPV2-085 conserved hypothetical protein	100					
PEPV2-093	97585-97334	SWPV2-086	83	83	SWPV2-086 HT motif protein	100				E	
PEPV2-094	98440-97982	SWPV2-087	152	146	SWPV2-087 conserved hypothetical protein	96.05				E	
PEPV2-095	99345-98542	SWPV2-088	267	267	SWPV2-088 virion protein	100				E	
PEPV2-096	99420-100247	SWPV2-089	275	275	SWPV2-089 T10-like protein	100					
PEPV2-097	100392-100255	SWPV2-090	45	45	SWPV2-090 conserved hypothetical protein	100					
PEPV2-098	100631-100374	SWPV2-091	85	85	SWPV2-091 ubiquitin	100					
PEPV2-099	101834-100857	SWPV2-092	325	339	SWPV2-092 conserved hypothetical protein	95.87					
PEPV2-100	102104-101862	SWPV2-093	80	80	SWPV2-093 hypothetical protein	100					
PEPV2-101	102697-102110	SWPV2-094	195	195	SWPV2-094 beta-NGF-like protein	100					
PEPV2-102	103227-102721	SWPV2-095	168	168	SWPV2-095 putative interleukin binding protein	100					
PEPV2-103	103539-103282	SWPV2-096	85	85	SWPV2-096 hypothetical protein	100					
PEPV2-104	103867-103550	SWPV2-097	105	105	SWPV2-097 conserved hypothetical protein	100					
PEPV2-105	104456-103884	SWPV2-098	190	190	SWPV2-098 N1R/p28-like protein	100					
PEPV2-106	104658-105035	SWPV2-099	125	125	SWPV2-099 putative glutaredoxin 2, virion morphogenesis	100	31.93	G4L	L	L	
PEPV2-107	105682-104978	SWPV2-100	234	234	SWPV2-100 putative elongation factor	100					
PEPV2-108	105676-105984	SWPV2-101	102	102	SWPV2-101 conserved hypothetical protein	100				L	
PEPV2-109	106120-106353	SWPV2-102	77	77	SWPV2-102 hypothetical protein	100				L	
PEPV2-110	106598-108496	SWPV2-103	632	632	SWPV2-103 putative metalloprotease, virion morphogenesis	100	43.85	G1L	E/L	E/L	
PEPV2-111	110525-108480	SWPV2-104	681	681	SWPV2-104 NPH-II, RNA helicase	100	43.79	I8R	L		
PEPV2-112	110560-111828	SWPV2-105	422	422	SWPV2-105 virion core proteinase	100	55.32	I7L	E/L	L	

PEPV2 synteny	PEPV2 genome coordinates	SWPV2 synteny	PEPV2 AA size	SWPV2 AA size	SWPV2 BLAST hits	PEPV2 AA identity (%)	PEPV2 AA identity compare to VACV-Cop (%)	VACV-Cop synteny	Predicted promoter in VACV-Cop	Predicted promoter in PEPV2	notes
PEPV2-113	111833-113008	SWPV2-106	391	391	SWPV2-106 DNA-binding protein	100	34.77	I6L	E/L		
PEPV2-114	113009-113254	SWPV2-107	81	81	SWPV2-107 putative IMV membrane protein	100				L	
PEPV2-115	113276-113815	SWPV2-108	179	179	SWPV2-108 thymidine kinase	100	51.96	J2R	E	L	
PEPV2-116	113936-114184	SWPV2-109	82	82	SWPV2-109 HT motif protein	100				E	
PEPV2-117	114254-115123	SWPV2-110	289	289	SWPV2-110 DNA-binding phosphoprotein	99.31	30.72	I3L	E		
PEPV2-118	115124-115333	SWPV2-111	69	69	SWPV2-111 conserved hypothetical protein	100				L	
PEPV2-119	115340-116272	SWPV2-112	310	310	SWPV2-112 DNA-binding virion protein	100	57.98	I1L	L	E/L	
PEPV2-120	116452-118410	SWPV2-113	652	652	SWPV2-113 conserved hypothetical protein	100	20.82	O1L	E		
PEPV2-121	118340-118735	SWPV2-114	131	131	SWPV2-114 virion core protein	100	34.85	E11L	L	L	
PEPV2-122	119013-118732	SWPV2-115	93	93	SWPV2-115 putative IMV redox protein, virus assembly	100	51.58	E10R	L		
PEPV2-123	119040-122006	SWPV2-116	988	988	SWPV2-116 DNA polymerase	100	50.30	E9L	E/L		
PEPV2-124	122825-121998		275			80.36	48.15	E8R	L		SWPV1-111 putative membrane protein
PEPV2-125	124542-122827	SWPV2-117	571	502	SWPV2-117 conserved hypothetical protein	99.80	49.82	E6R	E/L		
PEPV2-126	130342-124604	SWPV2-118	1912	1916	SWPV2-118 variola B22R-like protein	99.79					
PEPV2-127	135712-130409	SWPV2-119	1767	1767	SWPV2-119 variola B22R-like protein	100					
PEPV2-128	141691-135992	SWPV2-120	1899	1839	SWPV2-120 variola B22R-like protein	99.56					
PEPV2-129	141892-142440	SWPV2-122	182	182	SWPV2-122 RNA polymerase subunit RPO30	100	56.98	E4L	E/L		
PEPV2-130	142472-144637	SWPV2-123	721	721	SWPV2-123 conserved hypothetical protein	100	27.91	E2L	E/L		
PEPV2-131	144630-146048	SWPV2-124	472	472	SWPV2-124 poly(A) polymerase large subunit PAPL	100	50.54	E1L	L		
PEPV2-132	146401-146042	SWPV2-125	119	119	SWPV2-125 DNA-binding virion core protein	100	39.81	F17R	E/L		
PEPV2-133	146477-147100	SWPV2-126	207	207	SWPV2-126 conserved hypothetical protein	100				L	
PEPV2-134	147194-147640	SWPV2-127	148	148	SWPV2-127 conserved hypothetical protein	100	40.41	F15L	E/L		

PEPV2 synteny	PEPV2 genome coordinates	SWPV2 synteny	PEPV2 AA size	SWPV2 AA size	SWPV2 BLAST hits	PEPV2 AA identity (%)	PEPV2 AA identity compare to VACV-Cop (%)	VACV-Cop synteny	Predicted promoter in VACV-Cop	Predicted promoter in PEPV2	notes
PEPV2-135	147874-148173	SWPV2-128	99	99	SWPV2-128 conserved hypothetical protein	100					
PEPV2-136	153682-148244	SWPV2-129	1812	1801	SWPV2-129 variola B22R-like protein	99.39				E	
PEPV2-137	153832-154968	SWPV2-130	378	378	SWPV2-130 putative palmitylated EEV envelope lipase	100	38.01	F13L	L		
PEPV2-138	155046-156923	SWPV2-131	625	625	SWPV2-131 putative EEV maturation protein	100	26.34	F12L	E/L		
PEPV2-139	156966-158354	SWPV2-132	462	462	SWPV2-132 conserved hypothetical protein	100	27.05	F11L	E		
PEPV2-140	158445-159779	SWPV2-133	444	444	SWPV2-133 putative serine/threonine protein kinase, virus assembly	100	53.59	F10L	L	L	
PEPV2-141	159754-160395	SWPV2-134	213	213	SWPV2-134 conserved hypothetical protein	100	32.24	F9L	L		
PEPV2-142	160478-160678	SWPV2-135	66	66	SWPV2-135 conserved hypothetical protein	100					
PEPV2-143	161004-161558	SWPV2-136	184	184	SWPV2-136 HAL3-like domain protein	100					
PEPV2-144	161819-162784	SWPV2-137	321	321	SWPV2-137 N1R/p28-like protein	100					
PEPV2-145	162896-164911	SWPV2-138	671	671	SWPV2-138 ankyrin repeat protein	100	20.63	B4R	L		
PEPV2-146	164937-166607	SWPV2-139	556	556	SWPV2-139 ankyrin repeat protein	100	26.14	B4R	L		
PEPV2-147	166828-168150	SWPV2-140	440	440	SWPV2-140 conserved hypothetical protein	100	33.56	G5R	E		
PEPV2-148	168158-168346	SWPV2-141	62	62	SWPV2-141 RNA polymerase subunit RPO7	98.39	55.17	G5.5R	E		
PEPV2-149	168339-168905	SWPV2-142	188	188	SWPV2-142 conserved hypothetical protein	100	31.74	G6R	L		
PEPV2-150	169916-168870	SWPV2-143	348	348	SWPV2-143 virion core protein	100	35.66	G7L	L		
PEPV2-151	171002-170082	SWPV2-144	306	306	SWPV2-144 putative thioredoxin binding protein	100					
PEPV2-152	171130-171363	SWPV2-145	77	412	SWPV2-145 ankyrin repeat protein	100				E	
PEPV2-153	172717-171479	SWPV2-145	412	412	SWPV2-145 ankyrin repeat protein	100	41.86	M1L	E		
PEPV2-154	173393-172944	SWPV2-146	149	149	SWPV2-146 hypothetical protein	100					
PEPV2-155	174559-173621	SWPV2-147	312	312	SWPV2-147 Rep-like protein	100					
PEPV2-156	180803-174993	SWPV2-148	1936	875	SWPV2-148 variola B22R-like protein	98.20				E	
PEPV2-157	186333-180856	SWPV2-149	1825	1831	SWPV2-149 variola B22R-like protein	99.67					

PEPV2 synteny	PEPV2 genome coordinates	SWPV2 synteny	PEPV2 AA size	SWPV2 AA size	SWPV2 BLAST hits	PEPV2 AA identity (%)	PEPV2 AA identity compare to VACV-Cop (%)	VACV-Cop synteny	Predicted promoter in VACV-Cop	Predicted promoter in PEPV2	notes
PEPV2-158	186650-189133	SWPV2-150	827	834	SWPV2-150 hypothetical protein	89.06				E	
PEPV2-159	190261-189230	SWPV2-151	343	343	SWPV2-151 TGF-beta-like protein	100				E	
PEPV2-160	190263-190742	SWPV2-153	159	149	SWPV2-153 TGF-beta-like protein	41.27	30.56	D1R	E/L		
PEPV2-161	191211-191717	SWPV2-150	168	834	SWPV2-150 hypothetical protein	47.75					
PEPV2-162	192187-193200	SWPV2-154	337	320	SWPV2-154 N1R/p28-like protein	73.05					
PEPV2-163	194387-193299	SWPV2-152	362	358	SWPV2-152 TGF-beta-like protein	98.90				E	
PEPV2-164	194437-194886	SWPV2-153	149	149	SWPV2-153 TGF-beta-like protein	100					
PEPV2-165	195222-195530	SWPV2-154	102	320	SWPV2-154 N1R/p28-like protein	100					
PEPV2-166	195706-196299	SWPV2-154	197	320	SWPV2-154 N1R/p28-like protein	92.39				E	
PEPV2-167	196534-197571	SWPV2-155	345	345	SWPV2-155 Ig-like domain protein	99.71					
PEPV2-168	197843-198349	SWPV2-156	168	168	SWPV2-156 Ig-like domain protein	96.43				E	
PEPV2-169	198444-199577	SWPV2-157	377	350	SWPV2-157 N1R/p28-like protein	94.18					
PEPV2-170	200006-200644	SWPV2-158	212	212	SWPV2-158 thymidylate kinase	99.53	45.24	A48R	E/L		
PEPV2-171	200697-201479	SWPV2-159	260	260	SWPV2-159 late transcription factor VLTF-1	100	66.15	G8R	L	I/L	
PEPV2-172	201493-202500	SWPV2-160	335	335	SWPV2-160 putative myristylated protein	100	37.32	G9R	L	L	
PEPV2-173	202501-203232	SWPV2-161	243	243	SWPV2-161 putative myristylated IMV envelope protein	100	54.73	L1R	L	L	
PEPV2-174	203292-203582	SWPV2-162	96	96	SWPV2-162 conserved hypothetical protein	100					
PEPV2-175	204483-203572	SWPV2-163	303	303	SWPV2-163 conserved hypothetical protein	100	42.00	L3L	L		
PEPV2-176	204509-205267	SWPV2-164	252	252	SWPV2-164 DNA-binding virion core protein	100	36.25	L4R	L	L	
PEPV2-177	205268-205660	SWPV2-165	130	130	SWPV2-165 conserved hypothetical protein	100	41.59	L5R	L	L	
PEPV2-178	205614-206060	SWPV2-166	148	148	SWPV2-166 putative IMV membrane protein	100	42.47	J1R	L	L	
PEPV2-179	206094-207002	SWPV2-167	302	302	SWPV2-167 poly(A) polymerase small subunit PAPS	100	55.96	J3R	E/L		

PEPV2 synteny	PEPV2 genome coordinates	SWPV2 synteny	PEPV2 AA size	SWPV2 AA size	SWPV2 BLAST hits	PEPV2 AA identity (%)	PEPV2 AA identity compare to VACV-Cop (%)	VACV-Cop synteny	Predicted promoter in VACV-Cop	Predicted promoter in PEPV2	notes
PEPV2-180	206999-207559	SWPV2-168	186	186	SWPV2-168 RNA polymerase subunit RPO22	100	54.95	J4R	E		
PEPV2-181	207962-207552	SWPV2-169	136	136	SWPV2-169 conserved hypothetical protein	100	48.28	J5L	E/L		
PEPV2-182	208005-211871	SWPV2-170	1288	1288	SWPV2-170 RNA polymerase subunit RPO147	100	69.92	J6R	E	L	
PEPV2-183	212374-211874	SWPV2-171	166	166	SWPV2-171 putative protein-tyrosine phosphatase, virus assembly	100	47.62	H1L	L		
PEPV2-184	212390-212959	SWPV2-172	189	189	SWPV2-172 conserved hypothetical protein	100	48.91	H2R	L		
PEPV2-185	214021-213035	SWPV2-173	328	328	SWPV2-173 ankyrin repeat protein	100					
PEPV2-186	215056-214064	SWPV2-174	330	330	SWPV2-174 putative IMV envelope protein	100	32.20	H3L	E/L	E	
PEPV2-187	217547-215148	SWPV2-175	799	799	SWPV2-175 RNA polymerase associated protein RAP94	100	55.21	H4L	E/L		
PEPV2-188	217716-218228	SWPV2-176	170	170	SWPV2-176 late transcription factor VLTF-4	100					
PEPV2-189	218229-219179	SWPV2-177	316	316	SWPV2-177 DNA topoisomerase	100	57.98	H6R	L	L	
PEPV2-190	219184-219645	SWPV2-178	153	153	SWPV2-178 conserved hypothetical protein	100	33.78	H7R	L	L	
PEPV2-191	219919-219608	SWPV2-179	103	103	SWPV2-179 conserved hypothetical protein	100					
PEPV2-192	219927-222467	SWPV2-180	846	846	SWPV2-180 mRNA capping enzyme large subunit	100	53.86	D1R	E/L	L	
PEPV2-193	222538-222858	SWPV2-181	106	106	SWPV2-181 HT motif protein	100				L	
PEPV2-194	223277-222855	SWPV2-182	140	140	SWPV2-182 virion protein	100					
PEPV2-195	223331-223765	SWPV2-183	144	144	SWPV2-183 hypothetical protein	100					
PEPV2-196	223830-224402	SWPV2-184	190	190	SWPV2-184 conserved hypothetical protein	100				E	
PEPV2-197	224468-225295	SWPV2-185	275	275	SWPV2-185 N1R/p28-like protein	100				E	
PEPV2-198	225832-225362	SWPV2-186	156	156	SWPV2-186 C-type lectin-like protein	100	31.34	A34R	E/L		
PEPV2-199	226140-226817	SWPV2-187	225	225	SWPV2-187 deoxycytidine kinase-like protein	100					
PEPV2-200	226823-227323	SWPV2-188	166	166	SWPV2-188 Rep-like protein	99.40				E	
PEPV2-201	227382-227885	SWPV2-189	167	167	SWPV2-189 conserved hypothetical protein	100				E	

PEPV2 synteny	PEPV2 genome coordinates	SWPV2 synteny	PEPV2 AA size	SWPV2 AA size	SWPV2 BLAST hits	PEPV2 AA identity (%)	PEPV2 AA identity compare to VACV-Cop (%)	VACV-Cop synteny	Predicted promoter in VACV-Cop	Predicted promoter in PEPV2	notes
PEPV2-202	227939-228769	SWPV2-190	276	276	SWPV2-190 N1R/p28-like protein	100				E	
PEPV2-203	228842-229990	SWPV2-191	382	382	SWPV2-191 N1R/p28-like protein	100				E	
PEPV2-204	230046-230231	SWPV2-192	61	61	SWPV2-192 conserved hypothetical protein	100				L	
PEPV2-205	230450-231406	SWPV2-193	318	318	SWPV2-193 N1R/p28-like protein	100				L	
PEPV2-206	231467-232885	SWPV2-194	472	472	SWPV2-194 putative photolyase	100					
PEPV2-207	232942-233097		51								hypothetical protein, unique to PEPV2, detected 1 transmembrane helix
PEPV2-208	233075-233596	SWPV2-195	173	173	SWPV2-195 N1R/p28-like protein	100					
PEPV2-209	233702-234241	SWPV2-196	179	200	SWPV2-196 conserved hypothetical protein	89				L	
PEPV2-210	234285-235217	SWPV2-197	310	310	SWPV2-197 N1R/p28-like protein	100					
PEPV2-211	235265-235660	SWPV2-198	131	131	SWPV2-198 N1R/p28-like protein	100					
PEPV2-212	235715-235879	SWPV2-199	54	54	SWPV2-199 conserved hypothetical protein	100				L	
PEPV2-213	235939-236469	SWPV2-200	176	176	SWPV2-200 N1R/p28-like protein	100				L	
PEPV2-214	237180-236530	SWPV2-201	216	216	SWPV2-201 deoxycytidine kinase-like protein	100					
PEPV2-215	237354-238424	SWPV2-202	356	356	SWPV2-202 vaccinia C4L/C10L-like protein	100	28.11	C10L	E		
PEPV2-216	238699-239313	SWPV2-203	204	204	SWPV2-203 CC chemokine-like protein	100					
PEPV2-217	239403-240608	SWPV2-204	401	401	SWPV2-204 conserved hypothetical protein	100					
PEPV2-218	240722-241714	SWPV2-205	330	330	SWPV2-205 N1R/p28-like protein	100				E	
PEPV2-219	241802-243073	SWPV2-206	423	223	SWPV2-206 N1R/p28-like protein	99.53					
PEPV2-220	243313-243137		58								hypothetical protein, unique to PEPV2, detected 1 transmembrane helix
PEPV2-221	243531-243376		51								hypothetical protein, unique to PEPV2, detected 1 transmembrane helix
PEPV2-222	243881-244930	SWPV2-207	349	349	SWPV2-207 N1R/p28-like protein	100				E	

PEPV2 synteny	PEPV2 genome coordinates	SWPV2 synteny	PEPV2 AA size	SWPV2 AA size	SWPV2 BLAST hits	PEPV2 AA identity (%)	PEPV2 AA identity compare to VACV-Cop (%)	VACV-Cop synteny	Predicted promoter in VACV-Cop	Predicted promoter in PEPV2	notes
PEPV2-223	244984-245883	SWPV2-207	299	349	SWPV2-207 N1R/p28-like protein	73.22					
PEPV2-224	246476-246273	SWPV2-208	67	85	SWPV2-208 N1R/p28-like protein	62.71				E	
PEPV2-225	246924-247112	SWPV2-209	62	213	SWPV2-209 N1R/p28-like protein	100					
PEPV2-226	247580-248437	SWPV2-210	285	285	SWPV2-210 N1R/p28-like protein	100					
PEPV2-227	251499-248956	SWPV2-211	847	847	SWPV2-211 ankyrin repeat protein	99.65	31.55	B4R	L		
PEPV2-228	251753-252472	SWPV2-212	239	239	SWPV2-212 hypothetical protein	100					
PEPV2-229	252472-253206	SWPV2-214	244	126	SWPV2-214 N1R/p28-like protein	96.43					
PEPV2-230	254963-253659	SWPV2-215	434	434	SWPV2-215 ankyrin repeat protein	100	27.27	B4R	L		
PEPV2-231	255161-255358	SWPV2-216	65	65	SWPV2-216 hypothetical protein	100					
PEPV2-232	255306-255782	SWPV2-217	158	158	SWPV2-217 MyD116-like domain protein	100					
PEPV2-233	255812-256426	SWPV2-218	204	204	SWPV2-218 CC chemokine-like protein	94.61				E	
PEPV2-234	256608-258023	SWPV2-219	471	471	SWPV2-219 ankyrin repeat protein	100	30.39	M1L	E		
PEPV2-235	258043-259569	SWPV2-220	508	508	SWPV2-220 ankyrin repeat protein	100	30.53	M1L	E	E	
PEPV2-236	259640-260944	SWPV2-221	434	432	SWPV2-221 conserved hypothetical protein	99.54					
PEPV2-237	260989-261960	SWPV2-222	323	323	SWPV2-222 ribonucleotide reductase small subunit	100	70.94	F4L	E	E	
PEPV2-238	262141-263466	SWPV2-223	441	441	SWPV2-223 ankyrin repeat protein	99.55	33.33	B4R	L		
PEPV2-239	264202-263525	SWPV2-224	225	225	SWPV2-224 late transcription factor VLTF-3	100	76.68	A2L	L		
PEPV2-240	264417-264190	SWPV2-225	75	75	SWPV2-225 virion redox protein	100					
PEPV2-241	266410-264431	SWPV2-226	659	659	SWPV2-226 virion core protein P4b	100	54.14	A3L	L		
PEPV2-242	267144-266497	SWPV2-227	215	215	SWPV2-227 immunodominant virion protein	100					
PEPV2-243	267183-267692	SWPV2-228	169	169	SWPV2-228 RNA polymerase subunit RPO19	100	55.24	A5R	E/L	L	
PEPV2-244	268808-267687	SWPV2-229	373	373	SWPV2-229 conserved hypothetical protein	100	39.32	A6L	L		
PEPV2-245	270944-268815	SWPV2-230	709	709	SWPV2-230 early transcription factor large subunit VETFL	100	59.58	A7L	L		

PEPV2 synteny	PEPV2 genome coordinates	SWPV2 synteny	PEPV2 AA size	SWPV2 AA size	SWPV2 BLAST hits	PEPV2 AA identity (%)	PEPV2 AA identity compare to VACV-Cop (%)	VACV-Cop synteny	Predicted promoter in VACV-Cop	Predicted promoter in PEPV2	notes
PEPV2-246	271008-271910	SWPV2-231	300	300	SWPV2-231 intermediate transcription factor VITF-3	100	38.70	A8R	L		
PEPV2-247	272102-271875	SWPV2-232	75	75	SWPV2-232 putative IMV membrane protein	100					
PEPV2-248	274784-272103	SWPV2-233	893	893	SWPV2-233 virion core protein P4a	100	40.18	A10L	L		
PEPV2-249	274802-275641	SWPV2-234	279	279	SWPV2-234 conserved hypothetical protein	100	41.30	A11R	L	L	
PEPV2-250	276144-275638	SWPV2-235	168	168	SWPV2-235 virion protein	99.40	32.93	A12L	L		
PEPV2-251	276159-276416	SWPV2-236	85	56	SWPV2-236 conserved hypothetical protein	86.96					
PEPV2-252	276614-276405	SWPV2-237	69	69	SWPV2-237 putative IMV membrane protein	100					
PEPV2-253	276940-276662	SWPV2-238	92	92	SWPV2-238 putative IMV membrane protein	100	27.54	A14L	L		
PEPV2-254	277118-276957	SWPV2-239	53	53	SWPV2-239 putative IMV membrane virulence factor	100	35.29	A 14.5L	L		
PEPV2-255	277424-277134	SWPV2-240	96	96	SWPV2-240 conserved hypothetical protein	100					
PEPV2-256	278514-277408	SWPV2-241	368	368	SWPV2-241 predicted myristylated protein	100	39.44	A16L	L		
PEPV2-257	279108-278530	SWPV2-242	192	192	SWPV2-242 putative phosphorylated IMV membrane protein	100	34.57	A17L	E/L		
PEPV2-258	279126-280514	SWPV2-243	462	462	SWPV2-243 DNA helicase, transcriptional elongation	100	50.22	A18R	E/L		
PEPV2-259	280751-280482	SWPV2-244	89	89	SWPV2-244 conserved hypothetical protein	100	38.46	A19L	L		
PEPV2-260	281097-280759	SWPV2-245	112	112	SWPV2-245 conserved hypothetical protein	100	45.30	A21L	L		
PEPV2-261	281096-282400	SWPV2-246	434	434	SWPV2-246 DNA polymerase processivity factor	99.77	26.98	A20R	E/L		
PEPV2-262	282397-282855	SWPV2-247	152	152	SWPV2-247 Holliday junction resolvase protein	100	44.97	A22R	L		
PEPV2-263	282872-284023	SWPV2-248	383	383	SWPV2-248 intermediate transcription factor VITF-3	100	51.30	A23R	E		
PEPV2-264	284049-287522	SWPV2-249	1157	1157	SWPV2-249 RNA polymerase subunit RPO132	100	74.57	A24R	E/L		
PEPV2-265	289316-287511	SWPV2-250	601	601	SWPV2-250 A type inclusion-like protein	99.83					
PEPV2-266	290778-289351	SWPV2-251	475	475	SWPV2-251 A type inclusion-like/fusion protein	100	48.33	A27L	L		
PEPV2-267	291201-290779	SWPV2-252	140	140	SWPV2-252 conserved hypothetical protein	100	41.78	A28L	L		

PEPV2 synteny	PEPV2 genome coordinates	SWPV2 synteny	PEPV2 AA size	SWPV2 AA size	SWPV2 BLAST hits	PEPV2 AA identity (%)	PEPV2 AA identity compare to VACV-Cop (%)	VACV-Cop synteny	Predicted promoter in VACV-Cop	Predicted promoter in PEPV2	notes
PEPV2-268	292123-291206	SWPV2-253	305	305	SWPV2-253 RNA polymerase subunit RPO35	100	42.57	A29L	E		
PEPV2-269	292325-292098	SWPV2-254	75	75	SWPV2-254 conserved hypothetical protein	100					
PEPV2-270	292450-292791	SWPV2-255	113	113	SWPV2-255 conserved hypothetical protein	100	31.58	A31R			
PEPV2-271	292800-293162	SWPV2-256	120	120	SWPV2-256 conserved hypothetical protein	100				L	
PEPV2-272	294005-293151	SWPV2-257	284	284	SWPV2-257 DNA packaging protein	100	47.08	A32L	L		
PEPV2-273	294120-294665	SWPV2-258	181	181	SWPV2-258 C-type lectin-like EEV protein	100					
PEPV2-274	294890-295714	SWPV2-259	274	274	SWPV2-259 conserved hypothetical protein	100				E	
PEPV2-275	295774-296583	SWPV2-260	269	269	SWPV2-260 putative tyrosine protein kinase	100					
PEPV2-276	296626-297642	SWPV2-261	338	338	SWPV2-261 putative serpin	100					
PEPV2-277	298422-297664	SWPV2-262	252	252	SWPV2-262 conserved hypothetical protein	100					
PEPV2-278	298532-299464	SWPV2-263	310	310	SWPV2-263 G protein-coupled receptor-like protein	100					
PEPV2-279	299475-299765	SWPV2-264	96	96	SWPV2-264 conserved hypothetical protein	100				E	
PEPV2-280	299831-300361	SWPV2-265	176	169	SWPV2-265 beta-NGF-like protein	96.02					
PEPV2-281	300771-300379	SWPV2-266	130	130	SWPV2-266 HT motif protein	100					
PEPV2-282	300875-301519	SWPV2-267	214	214	SWPV2-267 conserved hypothetical protein	100					
PEPV2-283	301892-301530	SWPV2-268	120	120	SWPV2-268 HT motif protein	100					
PEPV2-284	302058-302393	SWPV2-269	111	111	SWPV2-269 CC chemokine-like protein	100					
PEPV2-285	302472-303053	SWPV2-270	193	193	SWPV2-270 putative interleukin binding protein	99.48					
PEPV2-286	303163-303543	SWPV2-271	126	126	SWPV2-271 EGF-like protein	100	34.78	C11R	E		
PEPV2-287	303545-304462	SWPV2-272	305	305	SWPV2-272 putative serine/threonine protein kinase	100	41.14	VPK1			
PEPV2-288	304505-304987	SWPV2-273	160	160	SWPV2-273 conserved hypothetical protein	100					
PEPV2-289	305070-305537	SWPV2-274	155	147	SWPV2-274 C-type lectin-like protein	99.28	22.56	A34R	L	L	
PEPV2-290	305580-305999	SWPV2-275	139	139	SWPV2-275 putative interleukin binding protein	100					

PEPV2 synteny	PEPV2 genome coordinates	SWPV2 synteny	PEPV2 AA size	SWPV2 AA size	SWPV2 BLAST hits	PEPV2 AA identity (%)	PEPV2 AA identity compare to VACV-Cop (%)	VACV-Cop synteny	Predicted promoter in VACV-Cop	Predicted promoter in PEPV2	notes
PEPV2-291	306068-306295	SWPV2-276	75	75	SWPV2-276 conserved hypothetical protein	100					
PEPV2-292	306497-308281	SWPV2-277	594	594	SWPV2-277 ankyrin repeat protein	99.83	22.28	B4R	L		
PEPV2-293	308305-308529	SWPV2-278	74	74	SWPV2-278 hypothetical protein	100					
PEPV2-294	308572-309426	SWPV2-279	284	284	SWPV2-279 ankyrin repeat protein	99.63	33.85	B24R / C18L			
PEPV2-295	309481-310773	SWPV2-280	430	430	SWPV2-280 ankyrin repeat protein	99.77	24.72	M1L	E		
PEPV2-296	310966-312156	SWPV2-281	396	396	SWPV2-281 ankyrin repeat protein	100	32.54	B4R	L		
PEPV2-297	312159-313535	SWPV2-282	458	458	SWPV2-282 ankyrin repeat protein	100	25.82	M1L	E	E	
PEPV2-298	313644-315857	SWPV2-283	737	737	SWPV2-283 ankyrin repeat protein	100	28.83	B4R	L		
PEPV2-299	315913-317628	SWPV2-284	571	571	SWPV2-284 ankyrin repeat protein	100	23.37	B4R	L	E	
PEPV2-300	317632-318534	SWPV2-285	300	300	SWPV2-285 putative serine/threonine protein kinase	99.67	32.76	B1R	E	E	
PEPV2-301	318607-319341	SWPV2-286	244	244	SWPV2-286 ankyrin repeat protein	100	23.70	M1L	E		
PEPV2-302	319942-321525	SWPV2-287	527	527	SWPV2-287 ankyrin repeat protein	100	25.19	B18R	E		
PEPV2-303	322121-321540	SWPV2-288	193	193	SWPV2-288 conserved hypothetical protein	100					
PEPV2-304	322189-323691	SWPV2-289	500	500	SWPV2-289 ankyrin repeat protein	100	28.30	M1L	E		
PEPV2-305	323907-325307	SWPV2-290	466	466	SWPV2-290 ankyrin repeat protein	100	27.65	B4R	L		
PEPV2-306	325378-326166	SWPV2-291	262	262	SWPV2-291 N1R/p28-like protein	100					
PEPV2-307	326228-326446	SWPV2-292	72	72	SWPV2-292 hypothetical protein	100					
PEPV2-308	326914-326450	SWPV2-293	154	154	SWPV2-293 C-type lectin-like protein	100	24.67	A34R	L		
PEPV2-309	327091-328164	SWPV2-294	357	357	SWPV2-294 ankyrin repeat protein	100					
PEPV2-310	328312-328902	SWPV2-295	196	196	SWPV2-295 ankyrin repeat protein	100	37.38	M1L	E		
PEPV2-311	329007-330620	SWPV2-296	537	537	SWPV2-296 ankyrin repeat protein	100					
PEPV2-312	330654-331028	SWPV2-297	124	124	SWPV2-297 EFc-like protein	100					
PEPV2-313	331038-331538	SWPV2-298	166	166	SWPV2-298 conserved hypothetical protein	100					

PEPV2 synteny	PEPV2 genome coordinates	SWPV2 synteny	PEPV2 AA size	SWPV2 AA size	SWPV2 BLAST hits	PEPV2 AA identity (%)	PEPV2 AA identity compare to VACV-Cop (%)	VACV-Cop synteny	Predicted promoter in VACV-Cop	Predicted promoter in PEPV2	notes
PEPV2-314	331610-332266	SWPV2-299	218	218	SWPV2-299 Ig-like domain protein	100					
PEPV2-315	332293-334182	SWPV2-300	629	629	SWPV2-300 ankyrin repeat protein	99.84	31.45	B4R	L	E	
PEPV2-316	334281-335228	SWPV2-301	315	315	SWPV2-301 G protein-coupled receptor-like protein	100					
PEPV2-317	335295-336929	SWPV2-302	544	544	SWPV2-302 ankyrin repeat protein	99.82	27.73	B4R	L		
PEPV2-318	337110-337277	SWPV2-303	55	55	SWPV2-303 hypothetical protein	100				E	
PEPV2-319	337452-338984	SWPV2-304	510	514	SWPV2-304 ankyrin repeat protein	99.22	36.84	M1L	E	E	
PEPV2-320	339248-341284	SWPV2-305	678	637	SWPV2-305 ankyrin repeat protein	99.53	25.79	M1L	E		
PEPV2-321	341476-342885	SWPV2-306	469	469	SWPV2-306 Ig-like domain protein	100				E	
PEPV2-322	343017-343391	SWPV2-307	124	124	SWPV2-307 EFc-like protein	100				E	
PEPV2-323	343725-345776	SWPV2-308	683	689	SWPV2-308 ankyrin repeat protein	100					
PEPV2-324	346536-346174	SWPV2-309	120	186	SWPV2-309 conserved hypothetical protein	70.83					identical to PEPV2-004
PEPV2-325	347140-346472	SWPV2-310	222	222	SWPV2-310 conserved hypothetical protein	100					identical to PEPV2-003
PEPV2-326	347548-348174	SWPV2-311	208	208	SWPV2-311 C-type lectin-like protein	100				E	identical to PEPV2-002
PEPV2-327	348992-348477	SWPV2-312	171	171	SWPV2-312 hypothetical protein	100				E	identical to PEPV2-001

Note: PEPV2, penguinpox virus 2; SWPV1, shearwaterpox virus 1; SWPV2, shearwaterpox virus 2; CNPV, canarypox virus; VACV-Cop, vaccinia virus Copenhagen strain; E, early; I, intermediate; L, late; %, percentage; Truncated or fragmented ORFs of PEPV2 compared to SWPV2 are highlighted in blue text.
